# Specific inhibition of the endothelin A receptor with ZD4054: clinical and pre-clinical evidence

**DOI:** 10.1038/sj.bjc.6602676

**Published:** 2005-06-14

**Authors:** C D Morris, A Rose, J Curwen, A M Hughes, D J Wilson, D J Webb

**Affiliations:** 1 AstraZeneca, Alderley Park, Macclesfield, Cheshire SK10 4TF, UK; 2 University of Edinburgh, Western General Hospital, Edinburgh, UK

**Keywords:** endothelin A receptor, receptor specificity, cancer, volunteer studies, ZD4054

## Abstract

Activation of the endothelin A receptor (ET_A_) by endothelin-1 (ET-1) mediates events that regulate mitogenesis, apoptosis, angiogenesis and metastasis in tumours. Specific blockade of ET_A_ may have anticancer effects, while retaining beneficial endothelin B receptor (ET_B_)-mediated effects such as apoptosis and clearance of ET-1. ZD4054 is an orally active, specific ET_A_ antagonist in clinical development. In receptor-binding studies, ZD4054 specifically bound to ET_A_ with high affinity; no binding was detected at ET_B_. In a randomised placebo-controlled trial in eight healthy volunteers, a single oral dose of ZD4054 reduced forearm vasoconstriction in response to brachial artery infusion of ET-1, thus providing clinical evidence of ET_A_ blockade. ET_B_ blockade was assessed in an ascending, single-dose, placebo-controlled trial in 28 volunteers. For all doses of ZD4054, mean plasma ET-1 concentrations measured at 4 and 24 h were within the placebo reference range (a rise in ET-1 would indicate ET_B_ blockade) and there was no evidence of dose-related changes. These data confirm the specificity of ZD4054 for ET_A_, with no activity at ET_B_ in a clinical or preclinical setting. As a result of this specificity, ZD4054 has the potential to block multiple ET_A_-induced pathological processes, while allowing beneficial ET_B_-mediated processes to continue, which may, in turn, lead to an effective cancer therapy.

There is accumulating evidence to suggest that endothelins, particularly endothelin-1 (ET-1), have a role in regulating the growth and proliferation of tumours (Nelson *et al*, 2003). ET-1, produced by tumour cells, exerts its effects primarily by binding to G-protein-coupled receptors on the cell surface (endothelin A receptor (ET_A_) and B receptor (ET_B_)) (Nelson *et al*, 2003) and modifying the effects of other growth factors (Nelson *et al*, 1995).

Binding of ET-1 to ET_A_ and ET_B_ causes distinct and opposing effects on cell growth and survival. In most cells, activation of ET_A_ promotes cell growth (Nelson *et al*, 2003), whereas activation of ET_B_ induces cell death via apoptosis (Okazawa *et al*, 1998). In addition, binding of ET-1 to ET_B_ results in clearance of ET-1 from the circulation. Overexpression of ET_A_ has been reported in a variety of human tumours and human cancer cell lines, including the prostate, ovary, lung, colon, kidney, cervix and bone (Nelson *et al*, 2003). Conversely, ET_B_ expression is reduced in the majority of solid tumours, but is still evident (Nelson *et al*, 2003). The balance of ET_A_ and ET_B_ activation in tumour cells appears to be important in progression of most cancers (Nelson *et al*, 2003), especially prostate cancer (Kopetz *et al*, 2002). Increased expression of ET_A_ relative to ET_B_ could contribute to increased tumour cell survival and growth.

Activation of ET_A_ by ET-1 is reported to result in a number of events involved in the malignant process, including regulating mitogenesis, apoptosis, angiogenesis and tumour metastasis. It triggers a signalling cascade involving growth factors such as epidermal growth factor and insulin-like growth factor-1 (Pirtskhalaishvili and Nelson, 2000), kinases including protein kinase C and mitogen-activated protein kinase (Bagnato *et al*, 1997; Bagnato and Catt, 1998), and induction of immediate-early response genes (*c-fos*, *c-jun* and *c-myc*) that promote cell growth and mitogenesis (Battistini *et al*, 1993). Additionally, apoptosis induced by cytotoxic agents is inhibited (Del Bufalo *et al*, 2002) and angiogenesis promoted (via a vascular endothelial growth factor (VEGF)-mediated mechanism) by activation of ET_A_ (Spinella *et al*, 2002; Bagnato and Spinella, 2003). The role of ET_A_ in mediating increased proliferation, resistance to apoptosis and survival of tumour cells, and increased angiogenesis – a process central to tumour growth – makes ET_A_ an attractive target for cancer therapy.

In addition to a role in growth and survival of primary tumours, ET_A_ is an attractive target to prevent the spread and survival of tumour metastases. Activation of ET_A_ induces the expression and activation of tumour proteases (matrix metalloproteinases and urokinase plasminogen activator) that facilitate tumour spread and metastasis (Rosanò *et al*, 2001). Furthermore, activation of ET_A_ leads to proliferation of osteoblasts, bone remodelling and release of growth factors that stimulate survival and growth of metastatic tumour cells (Nelson *et al*, 1999) within prostate cancer metastases in bone. These findings have led to extensive research into the endothelin receptors as a target for anticancer therapies.

Specific blockade of ET_A_ may offer an effective cancer therapy, since the anticancer effects of endothelin antagonists appear to be mediated via ET_A_ blockade. In contrast, antagonism of ET_B_ may lead to undesirable effects, such as inhibition of apoptosis and reduced clearance of ET-1. Thus, an agent with activity purely at the ET_A_ (i.e. a specific ET_A_ antagonist) would be desirable in an oncology setting. Atrasentan (Abbott Laboratories) is a selective ET_A_ antagonist currently in development which, while selectively binding to ET_A_, also exhibits antagonism of ET_B_ (Nelson, 2003), leading to increased plasma ET-1 levels (Carducci *et al*, 2002; Nelson, 2003). These findings are consistent with the binding affinities reported for atrasentan (0.034 and 63.3 nM for ET_A_ and ET_B_, respectively). ZD4054 (AstraZeneca) is an orally active ET_A_ antagonist in early clinical development for the treatment of cancer, which has recently been granted fast-track status by the FDA. The synthesis and molecular characterisation of ZD4054, a nonpeptide ET_A_ antagonist, has been described previously (Bradbury *et al*, 1997). ZD4054 binds to ET_A_ with high affinity and has no detectable affinity for ET_B_. In preclinical studies, ZD4054 specifically inhibits ET_A_-mediated antiapoptotic effects, but not ET_B_-mediated proapoptotic effects in human smooth muscle cells (Curtis *et al*, 2004; Dreicer *et al*, 2005), blocks ET_A_-mediated activation of p44/42 mitogen-activated protein kinase in murine osteoblast cells and inhibits ET-1 induced proliferation of human immature pre-osteoblast cells (Curtis *et al*, 2005). Importantly, ZD4054 inhibits growth of tumour xenografts in mice and enhances the cytotoxicity of paclitaxel in ovarian carcinoma *in vitro* and *in vivo* (Rosanò *et al*, 2005). This paper reports the results of studies that were conducted to confirm the specificity of ZD4054 for ET_A_ in a clinical setting.

## MATERIALS AND METHODS

### Receptor-binding assays

The inhibition by ZD4054 (varying concentrations) of ^125^iodine-ET-1 binding to cloned human ET_A_ and ET_B_ was assessed using standard radioligand-binding techniques. Human recombinant ET_A_ or ET_B_ was expressed in mouse erythroleukaemic cells, and cell membranes prepared for competitive binding studies using ^125^iodine-ET-1 as the radioligand. Incubations were carried out in triplicate in the presence of ZD4054, 100 pM to 100 *μ*M in half-log increments, and inhibition of ET-1 binding was expressed as the geometric mean pIC_50_ value (concentration to inhibit 50% of binding) with a 95% confidence interval (CI). The affinity of ZD4054 for cloned human ET_A_ was also assessed – using the equation of Cheng and Prusoff (1973) to determine the equilibrium dissociation constant (*K*_i_)– in a further receptor-binding screen utilising a greater number of concentration–response curves determined in three separate studies.

### Healthy volunteer study of forearm vasoconstriction to assess interaction with ET_A_

ET-1 causes vasoconstriction predominantly by activation of ET_A_ on vascular smooth muscle (Spratt *et al*, 2001). Therefore, inhibition of ET-1-induced vasoconstriction, measured by venous occlusion plethysmography (Wilkinson and Webb, 2001), would provide clinical evidence of ET_A_ blockade.

A single-dose, double-blind, placebo-controlled randomised trial was undertaken in eight healthy adult male volunteers to study the effect of ZD4054 on ET-1-mediated forearm vasoconstriction. All volunteers had previously demonstrated a mean 25–75% reduction in forearm blood flow (measured using standard strain gauge venous occlusion plethysmography) in response to a 120-min brachial artery infusion of ET-1. The effects of two oral doses of ZD4054 (10 and 30 mg) on ET-1-induced vasoconstriction were compared with placebo. Over nonconsecutive days, each volunteer received both doses of ZD4054 and placebo. The study was limited to two active doses and placebo due to the invasive nature and high technical difficulty of the brachial artery infusions and forearm vasoconstriction assessment. A 120-min brachial artery infusion of ET-1 (2.5 pmol min^−1^) was given to resting subjects, commencing 2 h after dosing with ZD4054 or placebo. The degree of forearm vasoconstriction measured between 90 and 120 min of the infusion (at 10-min intervals over a 30-min period) was compared between dose groups. The summary measure for statistical analyses was the percentage change in forearm blood flow. This measure was derived from the change from baseline (immediately prior to ET-1 infusion) in the mean area under the effect curve (forearm blood flow response) from 90 to 120 min (AUEC_90−120_) relative to the noninfused arm for each volunteer at each dose level *vs* placebo. Previous studies have shown that AUEC_90−120_ represents the most sensitive measure of ET_A_ antagonism as ET-1-induced vasoconstriction is usually maximal after 90 min (Strachan *et al*, 2002). Treatment and dose effects were compared using analysis of variance (ANOVA), fitting effects for subject and dose level.

### Healthy volunteer study of plasma ET-1 levels to assess interaction with ET_B_

A randomised, ascending, single-dose, double-blind, placebo-controlled study was undertaken in 28 healthy adult male volunteers. Oral doses of ZD4054 evaluated were 2.5, 10, 20, 30, 60, 120, 150 and 240 mg, with dose escalation continued based on tolerability until the maximum tolerated dose had been defined. The planned dose escalation sequence was from 120 to 240 mg ZD4054. However, the 240 mg dose was not tolerated; so the dose of 150 mg ZD4054 was investigated to further define the maximum tolerated dose. Volunteers were randomised approximately 3 : 1 to ZD4054 or placebo on each study day. Each cohort of volunteers was dosed consecutively on three separate study days, with a minimum of 14 days between doses in the same group. Doses of ZD4054 given were: group 1 (*n*=9) 2.5, 60 and 150 mg; group 2 (*n*=9) 10, 20 and 120 mg; group 3 (*n*=10) 30 and 240 mg. Blood samples were collected for measurement of plasma ET-1 (and its precursor, Big-ET-1), at baseline and at 4 and 24 h post-dose. An increase in ET-1 was taken as evidence of ET_B_ blockade. ET-1 and big ET were extracted from plasma using an acetic acid extraction technique described by Rolinski *et al* (1994). Concentrations of ET-1 and big ET-1 in the extract were determined by radioimmunoassay using a methodology based on commercially available assay kits (Peninsula Laboratories Inc., San Carlos, CA, USA). Briefly, 100 *μ*l of standard, sample or control was incubated with the appropriate antibody overnight. Samples were incubated with a known concentration of radio-labelled ET-1 or big ET-1 for a further 16 h and the immune complexes were precipitated with Amerlex™ (Amersham Plc, Amersham, UK) donkey anti-rabbit antibody. The sensitivities of the assays, defined as two standard deviations above the zero binding, were 0.25 pg ml^−1^ for ET-1 and 1 pg ml^−1^ for big ET-1.

Both clinical studies were approved by an Independent Ethics Committee and all subjects gave written informed consent. The study was performed in accordance with ethical principles originating in the Declaration of Helsinki and consistent with ICH/Good Clinical Practice, applicable regulatory requirements and AstraZeneca's policy on bioethics.

## RESULTS

### Receptor-binding assays

ZD4054 potently inhibited the binding of ^125^iodine-ET-1 to cloned human ET_A_ expressed in mouse erythroleukaemic cells, showing that ZD4054 has high affinity for ET_A_. The pIC_50_ for ZD4054 at the ET_A_ (geometric mean) was 8.27 nM (95% CI: 8.23, 8.32 nM) (*n*=4). Displacement curves were normal, with slopes close to unity. In the multi-receptor binding screen, pIC_50_ values for ZD4054 at ET_A_ were 22, 27 and 13 nM (mean value 21 nM) (Table 1). The *K*_i_ values measured in the same studies were 13, 17 and 8 nM (mean value 13 nM).

In contrast, ZD4054 had no measurable affinity for cloned human ET_B_, with a mean displacement of only 1.2±0.7% (*n*=3) of ^125^iodine-ET-1 at a concentration of 100 *μ*M ZD4054. This level of displacement is within the background range and is likely to be caused by assay variability. In the multi-receptor-binding screen, ZD4054 was inactive at ET_B_ at a concentration of 10 *μ*M (Table 1).

These data show that ZD4054 is a high-affinity ligand for ET_A_, with no measurable affinity for ET_B_.

### Healthy volunteer study of forearm vasoconstriction to assess interaction with ET_A_

**Figure 1:**
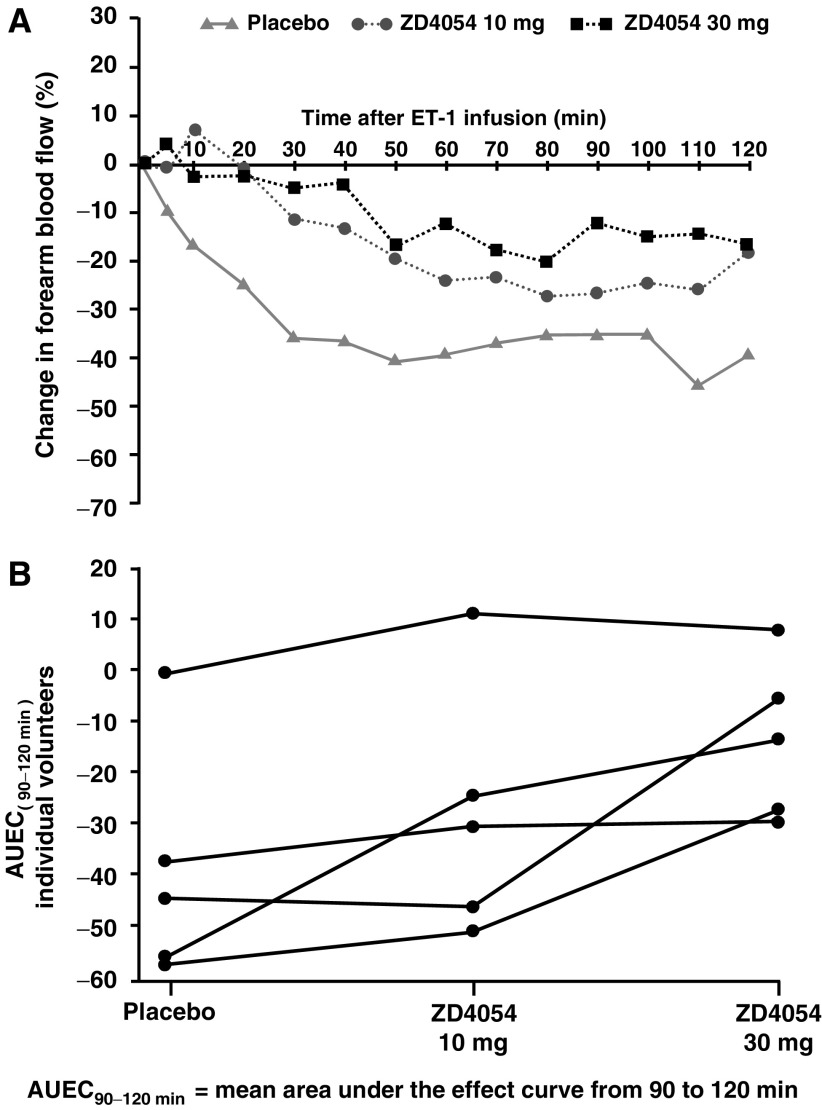
Administration of ZD4054 (10 and 30 mg) to healthy volunteers inhibits ET-1 induced vasoconstriction. (**A**) Mean change in forearm blood flow and (**B**) individual AUEC_(90−120 min)_ by dose level for five volunteers.

In volunteers given placebo, forearm blood flow was reduced by approximately 40% in response to brachial artery infusion of ET-1. ZD4054 inhibited this response to ET-1 (Figure 1A). The mean AUEC_(90−120 min)_ corresponding to the forearm blood flow for each dose and placebo is shown in Table 2 and the AUEC_(90−120 min)_ for individual patients at each dose level is shown in Figure 1B to illustrate the variability between individual volunteers. Administration of ZD4054 (10 or 30 mg; combined data) produced a statistically significant absolute reduction in vasoconstriction of 18.8% (*P*=0.021) when compared to placebo. Pairwise comparison showed that administration of ZD4054 (30 mg) produced a statistically significant absolute reduction in vasoconstriction of 23.7% (*P*=0.0125), representing a 63% decrease in vasoconstriction relative to placebo. ZD4054 (10 mg) resulted in a numerical decrease in vasoconstriction compared with placebo, which did not reach statistical significance (*P*=0.10). Peak plasma concentrations were reached by 1.75 and 2.5 h post-dosing and the mean plasma half-life of ZD4054 was 9.10 and 9.65 h for the 10 and 30 mg doses, respectively.

These results provide evidence that ZD4054 is an ET_A_ antagonist in healthy volunteers.

### Healthy volunteer study of plasma ET-1 levels to assess interaction with ET_B_

**Figure 2:**
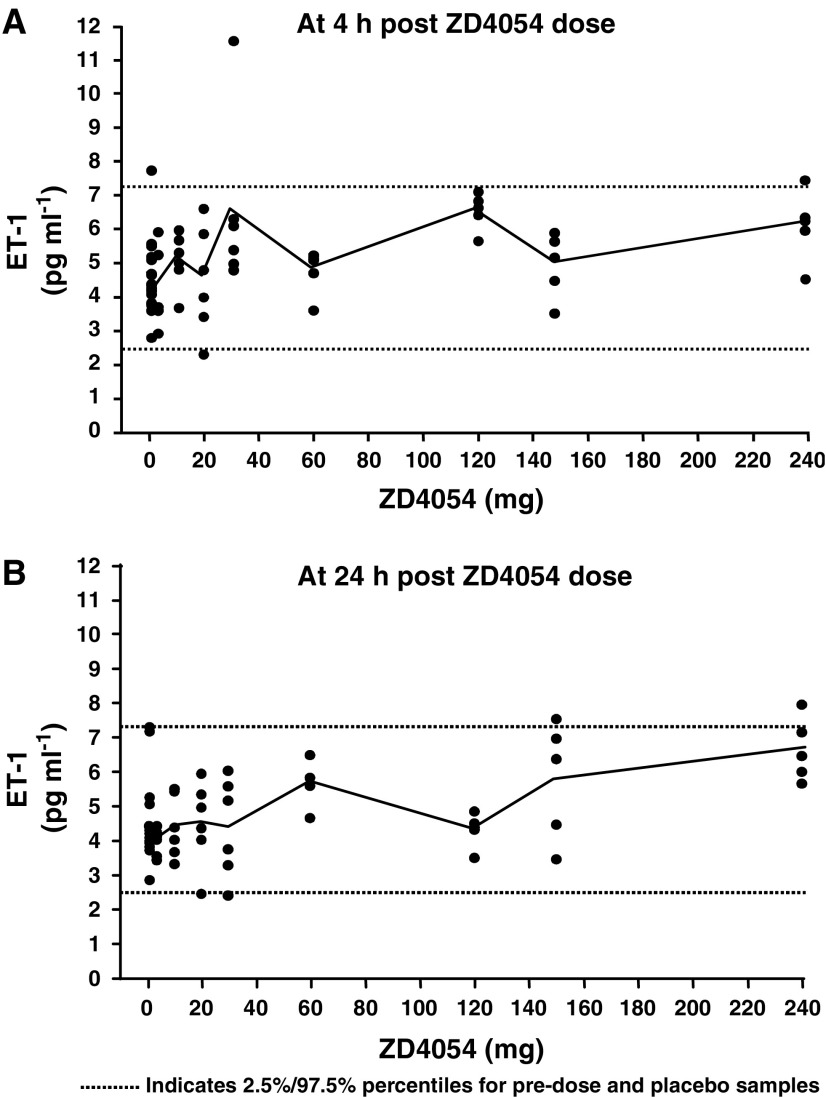
Administration of ZD4054 at doses upto 240 mg has no effect on plasma ET-1 concentrations in healthy volunteers at 4 (**A**) and 24 h (**B**) post-dose. Individual and mean data are shown.

Following administration of ZD4054 (2.5–240 mg), mean values for plasma ET-1 were within the placebo range at both 4 and 24 h post-dose (Figures 2A and B). The placebo range was defined by the 2.5 and 97.5% percentiles of the pre-dose and placebo (drug naïve) samples. Within this study, ZD4054 was well-tolerated at single doses up to and including 120 mg; dose escalation was limited by headache, nausea and vomiting. Based on a rise in mean ET-1 values (Figures 2A and B) or percentage change from baseline (data not shown), there was no evidence of a dose-related response across the 2.5–240 mg (twice the maximum well-tolerated dose) dose range tested. Since peak plasma concentrations of ZD4054 were reached by approximately 2 h post-dose, any impact on ET-1 clearance and plasma concentration of ET-1 can be expected to be detectable at 4 h post-dose. However, no consistent profile was observed when comparing the 4- and 24-h time points at each dose (Figures 2A or B). Similarly, there was no evidence of an increase in levels of Big ET-1, the precursor for ET-1 (data not shown).

These data, showing the inability of ZD4054 to alter plasma concentrations of ET-1 (a biomarker of ET_B_ blockade *in vivo* (Strachan *et al*, 1999)) in healthy volunteers, demonstrate the specificity of ZD4054 for ET_A_ in a clinical setting.

## DISCUSSION

Studies have shown that activation of ET_A_ by ET-1 results in a number of events that promote cell growth and mitogenesis (Battistini *et al*, 1993; Bagnato *et al*, 1997; Bagnato and Catt, 1998; Pirtskhalaishvili and Nelson, 2000), inhibit apoptosis induced by cytotoxic agents (Del Bufalo *et al*, 2002) and facilitate angiogenesis (Spinella *et al*, 2002; Bagnato and Spinella, 2003). Activation of ET_A_ by ET-1 also induces tumour proteases that facilitate tumour metastasis (Rosanò *et al*, 2001), and causes proliferation of osteoblasts, bone remodelling and release of growth factors that stimulate survival and growth of metastatic tumour cells (Nelson *et al*, 1999). As a result, ET_A_ is an attractive target for cancer therapy. Specific blockade of ET_A_ has the potential to mediate anticancer effects, while allowing beneficial effects such as apoptosis and clearance of ET-1 that are mediated by ET_B_ to proceed.

Results of the *in vitro* binding studies presented here show ZD4054 to be a potent and specific ET_A_ antagonist, exhibiting high-affinity binding to ET_A_, with no measurable affinity for ET_B_ at a concentration of 10 *μ*M. These results are consistent with previously reported molecular characterisation (Bradbury *et al*, 1997), and the results of functional assays showing that ZD4054 specifically inhibited ET_A_-mediated antiapoptotic effects, but not ET_B_-mediated proapoptotic effects, in human and rat smooth muscle cells (Curtis *et al*, 2004; Dreicer *et al*, 2005).

The experimental forearm vasoconstriction model is currently accepted as a standard technique for the investigation of vascular pharmacology and the impact of intra-arterial drug infusion in man (Wilkinson and Webb, 2001). Results using this model show ZD4054 to be a pharmacologically active ET_A_ antagonist, acting in a dose-related manner to reduce ET-1-induced vasoconstriction. This vasoconstriction is mediated primarily by ET-1 selective, vascular smooth muscle ET_A_ (Spratt *et al*, 2001). Although these results clearly demonstrate ET_A_ antagonism *in vivo*, they do not give any information regarding the affinity of ZD4054 for ET_B_. Thus, a further volunteer study was undertaken to explore the specificity of ZD4054 for ET_A_.

In healthy volunteers, the concentration of circulating ET-1 has been established as a biomarker of ET_B_ blockade *in vivo* (Strachan *et al*, 1999). In this setting, a rise in plasma ET-1, particularly without an accompanying rise in Big ET-1, indicates ET_B_ inhibition. In the healthy volunteer study reported here, no evidence of ZD4054-induced ET_B_ inhibition was detected; mean plasma levels of ET-1, at all doses of ZD4054, were within the placebo range at 4 and 24 h post-dose. No clinically significant rise in plasma ET-1 was observed when ZD4054 was given at doses up to 240 mg (twice the maximum tolerated dose). Furthermore, there was no evidence of a dose-related response based on a rise in mean ET-1 or percentage change from baseline. These data provide evidence that single doses of the ET_A_ antagonist ZD4054 do not inhibit clearance of ET-1, and therefore that ZD4054 does not inhibit ET_B_ in man. Through its specificity for ET_A_, ZD4054 may offer advantages over other less specific ET_A_ antagonists in the oncology setting. Any degree of binding to ET_B_ has the potential to reduce the efficacy of ET_A_ blockade strategies, both directly through inhibition of ET_B_-mediated apoptosis and indirectly by reduction of ET-1 clearance, leading to a rise in levels of the ET_A_ ligand, ET-1. Treatment with the selective ET_A_ antagonist atrasentan (10 mg once daily for 28 days) resulted in a significant increase in plasma ET-1 levels in a study of patients with refractory adenocarcinomas (Carducci *et al*, 2002). Plasma levels of ET-1 rose linearly with increasing dose of atrasentan (dose range evaluated, 10–75 mg). This increase in plasma levels of ET-1 suggests reduced clearance of ET-1, an effect that could impair the efficacy of any ET_A_-blocking strategy. The authors hypothesised that the rise in plasma ET-1 reported with atrasentan was the result of direct ET_A_ blockade (Carducci *et al*, 2002). Although it is difficult to extrapolate between patients and healthy volunteers, evidence from the present study shows that blockade of ET_A_ by ZD4054, which has no detectable affinity for ET_B_ (at a concentration of 10 *μ*M), does not result in elevated plasma levels of ET-1. Furthermore, the ability of atrasentan to increase plasma levels of ET-1 has been attributed to blockade of ET_B_ (Nelson, 2003) and suggests that the system is highly sensitive to ET_B_ blockade. To our knowledge, ZD4054 is the only endothelin receptor antagonist in clinical development that targets ET_A_ and does not inhibit ET_B_ at doses under clinical investigation.

In conclusion, volunteer studies and pre-clinical receptor-binding studies confirm that ZD4054 is a potent antagonist of ET_A_, with no evidence of ET_B_ blockade at doses upto 240 mg in volunteers and at 10 *μ*M *in vitro*. This lack of affinity for ET_B_ suggests that ZD4054 has the potential to block the multiple pathological processes in malignancy that are mediated by ET_A_, while allowing the beneficial processes mediated by ET_B_, such as apoptosis and clearance of ET-1, to proceed. Further studies to assess the clinical impact of specific ET_A_ inhibition by ZD4054 in patients with cancer are ongoing.

## Figures and Tables

**Table 1:** Effect of ZD4054 on the binding of ^125^I-ET-1 to cloned human ET_A_ and ET_B_

**ZD4054 binds specifically to ET_A_**		
	**pIC_50_**	**Standard error**
ET_A_	21 nM	±4
ET_B_	Not detected (>10 *μ*M)	

pIC_50_=concentration required to inhibit 50% of binding.

**Table 2:** Mean forearm blood flow (FBF) AUEC_(90–120 min)_

**Treatment**	***N***	**FBF AUEC _90–120 min_ mean (%)**	**90% CI**
Placebo	5	−39.5	−61.6, −17.4
ZD4054 (30 mg)	6	−14.7	−26.3, −3.0
ZD4054 (10 mg)	6	−24.5	−44.3, −4.7

ET-1 (2.5 pmol min^−1^) was administered over 2 h after treatment with placebo or ZD4054.

AUEC_90–120 min_=mean area under the effect curve from 90 to 120 min.
